# Monolithic integrated optoelectronic chip for vector force detection

**DOI:** 10.1038/s41378-024-00712-6

**Published:** 2024-06-24

**Authors:** Jiansong Feng, Zhongqi Wang, Mengyuan Zhanghu, Xu Zhang, Yong Shen, Jing Yang, Zhibin Li, Bin Chen, Taihong Wang, Xiaolong Chen, Zhaojun Liu

**Affiliations:** 1 Department of Electrical and Electronic Engineering, Southern University of Science and Technology, Shenzhen, 518055 China; 2 Department of Electrical and Electronic Engineering, The University of Hong Kong, Hong Kong, China

**Keywords:** Optical sensors, Electrical and electronic engineering

## Abstract

Sensors with a small footprint and real-time detection capabilities are crucial in robotic surgery and smart wearable equipment. Reducing device footprint while maintaining its high performance is a major challenge and a significant limitation to their development. Here, we proposed a monolithic integrated micro-scale sensor, which can be used for vector force detection. This sensor combines an optical source, four photodetectors, and a hemispherical silicone elastomer component on the same sapphire-based AlGaInP wafer. The chip-scale optical coupling is achieved by employing the laser lift-off techniques and the flip-chip bonding to a processed sapphire substrate. This hemispherical structure device can detect normal and shear forces as low as 1 mN within a measurement range of 0–220 mN for normal force and 0–15 mN for shear force. After packaging, the sensor is capable of detecting forces over a broader range, with measurement capabilities extending up to 10 N for normal forces and 0.2 N for shear forces. It has an accuracy of detecting a minimum normal force of 25 mN and a minimum shear force of 20 mN. Furthermore, this sensor has been validated to have a compact footprint of approximately 1.5 mm^2^, while maintaining high real-time response. We also demonstrate its promising potential by combining this sensor with fine surface texture perception in the fields of compact medical robot interaction and wearable devices.

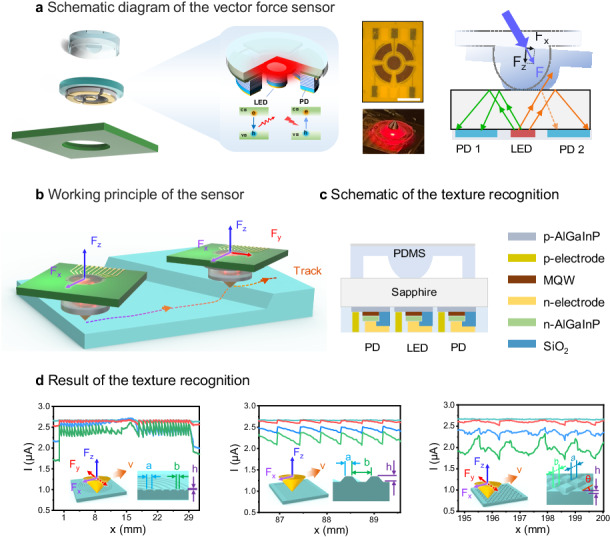

## Introduction

Human touch relies primarily on the soft skin and abundant sensory receptors in the fingertips to obtain information about the shape, texture, and hardness of objects [1, 2]. These features encompass various tactile information, such as vector forces (normal force and shear force), temperature, and roughness [3]. Among these, the measurement of spatial vector forces holds significant importance. For instance, when evaluating the movement of a finger and wrist motion with a force sensor, it is necessary the detection of force with magnitude and direction simultaneously [4]. Collecting both normal direction and shear direction force is essential in robotic hand-grasping tasks, especially when dealing with deformable objects [5]. With a shear force sensor, a robotic hand can handle objects in motion with minimal shear force, preventing damage to both the robotic hand and fragile objects, and increasing robot’s service life [6]. Therefore, a sensor with shear force detection capability is of great significance in obtaining accurate and rich pressure-sensing signals, as well as expanding the application scenarios of force sensors.

Recently, there have been numerous breakthroughs in force sensors with applications in emerging medical monitoring [7], robotic surgery [8, 9], and prosthetics [10, 11]. As a prominent type of sensor, it has been accomplished for various complex tasks [12–14]. They operate based on various mechanisms, including resistive [15, 16], piezoelectric [17], capacitive [18, 19], and optoelectronic [20, 21] sensors. Among them, resistive and capacitive sensor arrays are susceptible to crosstalk effects, requiring complex techniques to decouple the normal and shear forces. Pyroelectric effects exhibited by piezoelectric materials can affect sensor performance. Self-powered sensors are limited to dynamic force measurements [22]. In comparison, optical sensors have inherent advantages, such as good stability and unparalleled immunity to electromagnetic interference (EMI). However, research into such sensors is currently based on large and expensive external light sources and optical signal demodulation equipment, as well as the complex alignment and assembly of optical path coupling systems, presenting significant challenges for miniaturizing sensors needed in numerous application domains.

In detailed related studies, Xiu He utilized three orthogonally distributed optical fiber Fabry-Perot strain sensors to achieve three-dimensional force sensing [23]. The sensor showed good repeatability and promising application prospects. however, the system is complex due to the need for external light sources and costly optical signal demodulation equipment. Moreover, Artémis Llamosi designed a more simplified vector force sensor consisting of an array of silicone rubber (PDMS) force sensing units and an imaging system [24]. This sensor can measure three-dimensional forces ranging from 0 to 3 N. The use of a photodetector (PD) to measure light intensity, instead of relying on the spectrum, simplifies the system and eliminates the need for large spectrometer-type demodulation equipment. Duncan G. Raitt introduced a more compact three-dimensional force sensor that utilizes an endoscopic camera for multi-marker inspection of the inner surface of a flexible membrane. This sensor can measure forces up to 2.799 N with an accuracy of 0.110 N, even when the height of the membrane exceeds 20 mm [25]. Furthermore, Jingyi Zhou developed a very compact vector force sensor, consisting of a waveguide structure embedded in an organic silicone elastomer, with LEDs and PDs at each end [26]. The sensor achieved an average accuracy of 28.0 mN for normal forces and 81.1 mN for shear forces, both within a sensing range of 1 N. Although the sensor has been scaled down to dimensions of 23 mm in width and 3 mm in thickness, achieving further size reduction through conventional methods is challenging due to the limitations imposed by the size of commercial electronic components. Consequently, the current capabilities are insufficient to meet the demand for millimeter-scale vector force sensors required in fields such as surgical robotics and spacecraft [27].

**Fig. 1:**
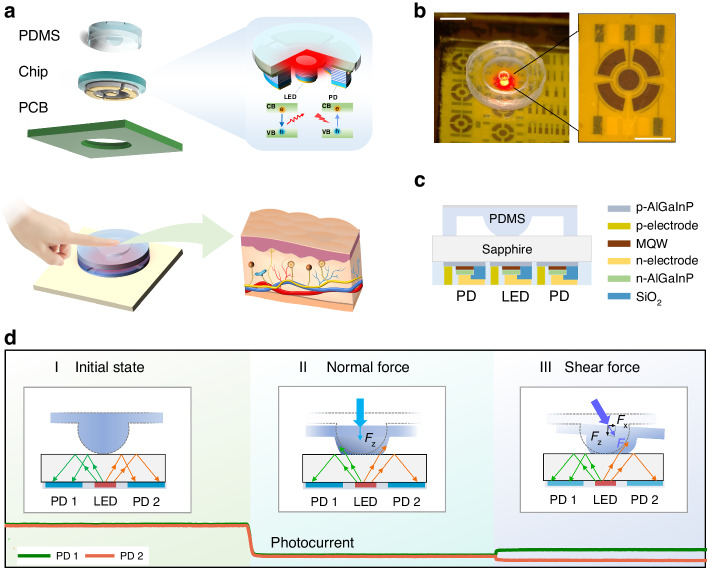
Schematic diagram of the vector force sensor. **a** Structure of the sensor, including the integrated optoelectronic chip, PCB, and a PDMS elastomer with a hemispherical structure. **b** Images of the fabricated devices. The scale bar in the image is 1 mm. **c** Cross-sectional diagram of the integrated sapphire-based AlGaInP chip. MQW is short for multi-quantum well. **d** Schematic diagram illustrating the working principle of the vector force sensor

To address these issues, we proposed a monolithic integrated micro-scale vector force sensor, capable of simultaneously detecting both the magnitude and direction of pressure. As shown in Fig. 1a, the sensor comprises three parts: micro-LED and PD devices for processing optoelectronic signals, a sapphire serving as the optical coupling medium, and a PDMS elastomer that senses external pressure. The sensor achieves a very compact structure by directly using the substrate of the chip as the optically coupling medium. Our selection of PDMS is attributed to its close resemblance to the Young’s modulus of human skin [28], allowing for the effective conversion of force into deformation. The entire device operates in the biologically safe red-light spectrum, enabling the sensor to be applied in biocompatible scenarios such as surgical robotics and Wearable Health Monitoring devices. Here, we chose the AlGaInP quantum well structure LED, which has the highest luminous efficiency in the red-light band among existing mature processes, as the light source. As we all know, the AlGaInP was growth on GaAs substrate. However, due to the absorption of red light by GaAs, it cannot be directly used as an optical coupling medium. Therefore, by employing the lift-off and bonding process, we replaced the GaAs substrate with a sapphire substrate that has higher transparency to red light. This ultimately achieved the monolithic integration of optoelectronic devices and optical coupling media.

The manufactured sensor features one micro-LED with a radius of 238 μm and four fan-shaped PDs arranged (each about 600 μm^2^) around it were integrated on the same AlGaInP wafer, which uses the same wafer-scale manufacturing process. The surface protruding hemispherical PDMS elastomer deforms under pressure, changing the reflective properties of the sapphire surface and subsequently modifying the light coupling to the four PDs distributed in different directions. Since the deformation of the PDMS elastomer is related not only to the magnitude but also to the direction of the force, the analysis of changes in the photocurrent of each PD in the four directions allows simultaneous measurement of the magnitude and direction of the force. Additionally, the sensitivity and measurement range of the system can be easily adjusted by changing the shape of the PDMS elastomer to suit different application scenarios. This hemispherical structure device is capable of detecting normal and shear forces as low as 1 mN within a measurement range of 0–220 mN for normal force and 0–15 mN for shear force. After packaging, the sensor’s range is expanded, enabling it to detect a minimum normal force of 25 mN and a minimum shear force of 20 mN, with a measurement range of 10 N for normal force and 0.2 N for shear force. Finally, we analyze the sensing mechanism of this device and its response to force changes in different directions, validating the performance of the proposed microsensor. It eliminates the need for large and expensive external light sources and optical signal demodulation equipment and features a small footprint, easy integration, high repeatability, fast response, biocompatible, and immunity to magnetic interference, making it suitable for a wide range of applications.

## Result

### Sensor design

A schematic layout of fully-integrated sensors is displayed in Fig. 1a, where the PDMS elastomer with the hemispherical structure attached to the optoelectronic chip, was mounted on a printed circuit board (PCB). The fabricated devices are shown in Fig. 1b. The central circular brown area represents the micro-LED, surrounded by four fan-shaped regions corresponding to the PDs, as shown in the enlarged diagram on the right. Figure 1c presents the cross-sectional schematic diagram of the integrated AlGaInP optoelectronic chip. The discrete devices were fabricated on the sapphire-based AlGaInP wafer, which served as the light-emitting micro-LED and the light-receiving PD, respectively.

The schematic diagram of the sensor working principle is shown in Fig. 1d. When current is injected into the multi-quantum wells (MQW) of the central micro-LED, radiative recombination of confined charge carriers in the MQW produces light emission. Subsequently, a portion of the emitted light undergoes total internal reflection (TIR) at the interface between the sapphire and air, then coupled into the surrounding PDs. Under external pressure loads, the hemispherical PDMS elastomer undergoes deformations and varies its contact area with the sapphire, leading to changes in the reflectance at the sapphire boundary and altering the amount of light reaching each PD. Detailed examples are provided in Fig. 1d. Without external force, as shown in regime I of Fig. 1d, PD 1 and PD 2 received constant light intensities. When normal pressure is applied, as shown in regime II of Fig. 1d, the PDMS elastomer undergoes isotropic compression deformation in the horizontal direction, causing the light rays at the interface between the sapphire and PDMS to scatter into the surrounding environment due to the change of boundary conditions. As a result, both PDs received a reduced amount of light, leading to a decrease in photocurrent. In regime III of Fig. 1d, when pressure with a horizontal shear force component is applied, the deformation of the PDMS elastomer is no longer uniform in the left and right directions, and the contact area between the PDMS elastomer and the sapphire exhibits asymmetry. Thus, the reflected light reduces in PD 2 and its photocurrent decreases (indicated by the orange line in Fig. 1d), while PD 1 receives more reflected light and its photocurrent increases (indicated by the green line in Fig. 1d).

Based on the same principle, we fabricated a chip with a central micro-LED and four surrounding PDs. When the pressure load is applied, the deformation of the PDMS microsphere caused by shear force is different in each direction, resulting in different changes in the contact area with the sapphire surface. The direction and magnitude of the normal and shear force were obtained by analyzing the changes in the photocurrent from four PDs. Thereby, simultaneous detection of force magnitude and direction could be achieved. Due to the easily deformable nature of the hemispherical PDMS elastomer structure under both shear and normal forces, it can realize multi-directional and real-time measurement sensors with highly sensitive and rapid responsiveness.

### Simulation design

Simulation analyses were conducted to evaluate the mechanical performance and optical performance of the device.

**Fig. 2:**
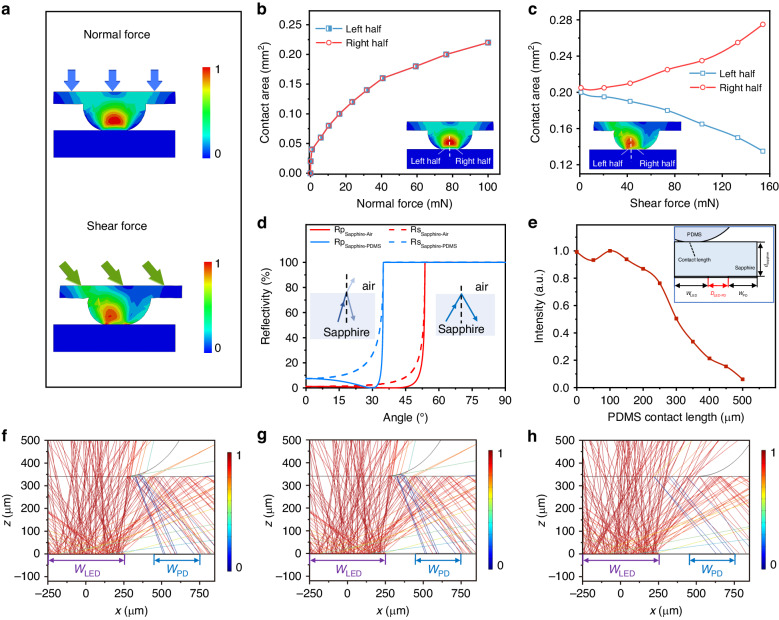
Simulation analyses results of device mechanical and optical performance. **a** Schematic diagram of the mechanical simulation model. Relationship between the contact length of PDMS and sapphire with respect to variations in the normal force (**b**) and shear force (**c**). **d** Calculation of the reflectivity at the sapphire/air and sapphire/PDMS interfaces as a function of angle. Ray tracing Simulation results: **e** Relationship between the PDMS contact length and the light received by the PD. Ray-tracing patterns for PDMS contact length of (**f**) 0 μm, (**g**) 250 μm, and (**h**) 500 μm with the sapphire

Using finite element analysis (FEA), we simulated the deformation of the hemisphere PDMS elastomer under normal and shear forces. Figure 2a illustrates the normalized strain distribution of the PDMS elastomer when subjected to normal and shear forces, with red indicating the highest stress. It is clearly observed that under normal force, the deformation is relatively symmetric on the left and right sides of the PDMS elastomer. As the normal force is increased, the contact length between the PDMS elastomer and the sapphire surface on both the left and right sides increases (see Fig. 2b). When shear force is applied, there is a noticeable asymmetry in the strain distribution. Specifically, when the shear force is applied in the rightward direction, the contact length on the right half side increases with the enhancement of the shear force, while the contact length on the left side decreases (see Fig. 2c). Therefore, external force causes changes in the contact between the PDMS elastomer and the sapphire surface, altering the boundary conditions and impacting light reflectance within the contact region.

According to the Fresnel reflection formula, we calculated the interface reflectance for the sapphire in external environments of either air or PDMS. The critical angles for total internal reflection at the sapphire/air interface and the sapphire/PDMS interface are 34.2° and 53.0°. As shown in Fig. 2d, light with incident angles greater than the critical angle undergoes TIR, while those with incident angles less than the critical angle typically exhibit reflectance below 10%. Since the micro-LED served as an omni-directional light source, we conducted a simulation using the ray tracing method to analyze the geometrical optical characteristics of light propagation from the micro-LED through the sapphire to the PD. The simulation results are shown in Fig. 2e. As the contact length between PDMS and sapphire increased, the light intensity received by the PD decreased. Figure 2f–h illustrates detailed ray tracing patterns for different contact lengths. With the increase in the contact length between PDMS and sapphire, the light intensity received by the PD diminished, aligning the geometric optics of the device with the functional requirements of the sensor.

### Characteristics of the integrated optoelectronic chip

**Fig. 3:**
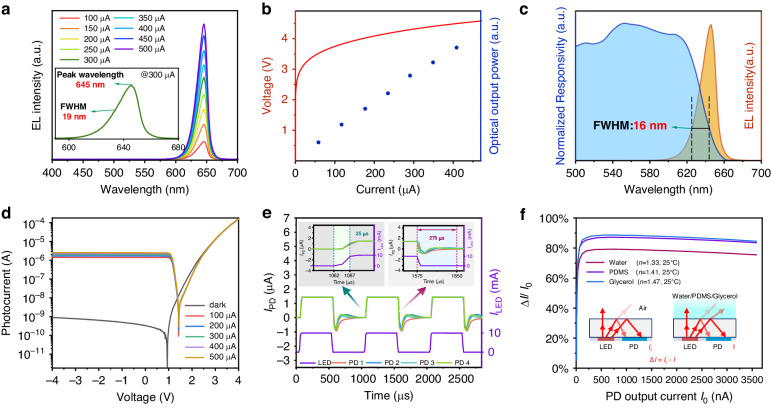
Optical and electrical properties of the AlGaInP device. **a** Room temperature EL spectra of the micro-LED measured under varying injection currents. **b** micro-LED current-voltage (I-V) characteristics. **c** EL spectrum of the micro-LED biased at 0.4 mA and normalized spectral response characteristics of the zero-bias PD. **d** I-V characteristics of the PD measured in darkness and illumination from the micro-LED with the same MQW-diode structure operated at varying currents. **e** The response speed of PD to light emitted by LEDs on the chip. **f** The relationship between the photocurrent variation on the sapphire surface of the chip covered with three different refractive index materials: water, PDMS, and glycerol

As the core component of the sensor, the performance of the integrated AlGaInP chip was characterized and analyzed. Firstly, the electroluminescence (EL) characterized were measured using a spectrometer (Maya2000Pro, Ocean Optics). Figure 3a displays the micro-LED’s EL spectrum for injection currents ranging from 50 μA to 500 μA, with the inset showing the spectrum at 300 μA. The EL emission peak is centered at ~646 nm with a full width at half maximum (FWHM) of ~19 nm. The current-voltage (I-V) characteristics of the micro-LED and the relative intensity of the emission peak at corresponding injection currents, as shown in Fig. 3b.

It was essential to investigate the ability of PD to detect the reflected light emitted from the micro-LED, which is critical in determining the performance of the integrated optoelectronic chip. The spectral responsivity of the PD was obtained by illuminating it with monochromatic light generated from a broadband visible light source combined with a monochromator (Omni-λ200i, Zolix). Figure 3c plots the EL spectrum of micro-LED biased at 0.4 mA and the normalized responsivity of the PD. The sensitivity of the PD decreased with increasing wavelengths, indicating a spectral overlap region with a FWHM of ~16 nm with the EL spectrum of the micro-LED. The photodetection performance of the PD was further studied by measuring its I-V characteristics using a source measurement unit (SMU) (NI Pxie 4135). as presented in Fig. 3d. Under dark conditions, the photocurrent of the PD measured at either reverse or zero bias voltage exhibited very low levels of ~10^−10^ A. However, when a current was injected into the micro-LED, the photocurrent of the PD significantly increased to the range of 10^−5^ to 10^−6^ A, and increased as the operation current of the micro-LED increased from 0.1 mA to 0.5 mA.

Subsequently, we conducted a study on the response time of the chip. The LED was driven by a square wave current signal at 1 kHz, and the variation of photocurrent in PD was observed. The photocurrent signal of PD was collected using an SMU with a sampling frequency of 80 kHz. As shown in Fig. 3e, the response characteristics of PD are synchronized with the LED driving current signal. The rising edge time of the photocurrent response in PD is 25 μs, and the falling edge time is 275 μs. There is fluctuation in the photocurrent at the falling edge, which is likely primarily due to parasitic capacitance effects in the device.

To determine the optimal operating current for the chip, we measured the changes in photocurrent before and after completely covering the sapphire surface of the chip with PDMS. As shown in Fig. 3f, when the sapphire surface is covered with PDMS, there is more than an 80% change in output photocurrent compared to when the sapphire surface is exposed to air. As the initial operating current of the chip increases, the change in PD photocurrent first increases rapidly and then decreases slowly. The maximum change occurs around an I_0_ of 600 nA, with a change of 87.2%. Considering the lower signal-to-noise ratio at lower current levels, we ultimately chose 1000 nA as the initial photocurrent value for the chip’s operation.

Additionally, a portion of light in the device directly radiates from the side of the LED to the PD without being reflected by the sapphire surface. This portion of the light is not affected by changes in the external refractive index and manifests as a constant output photocurrent in the device. It is necessary to analyze this portion of light. We covered the sapphire surface with liquids of different refractive indices and measured the changes in the PD’s photocurrent before and after the sapphire surface was covered, as shown in Fig. 3f. It can be observed that when the sapphire surface of the chip is covered with three different materials, the photocurrent decreases significantly. As the refractive index increases, the change in photocurrent also increases. When I_0_ is around 600 nA, the changes are 79.2% (water, *n* = 1.33), 87.2% (PDMS, *n* = 1.41), and 88.7% (glycerol, *n* = 1.47), respectively. This indicates that the photocurrent from the directly coupled light is below 10%, which has a minimal impact on the sensor’s performance.

### The vector force sensing characteristics of the hemispherical PDMS

The feasibility and response characteristics of using PDMS hemispherical structure in sensors were tested and analyzed. The test setup was presented in Supplement 1, Fig. S1. The hemisphere PDMS elastomer was fixed to the probe of a force gauge, and was aligned concentrically with the micro-LED in the horizontal direction. A three-dimensional (3D) translation stage was then used to control the hemisphere PDMS elastomer to approach the sapphire surface of the chip. Either normal or shear force was applied to the PDMS surface by adjusting the three-dimensional stage. Due to inevitable errors and defects in the fabrication process, there were certain differences in the photocurrents of the four PDs. Hence, the obtained photocurrents were normalized to facilitate the research analysis.

**Fig. 4:**
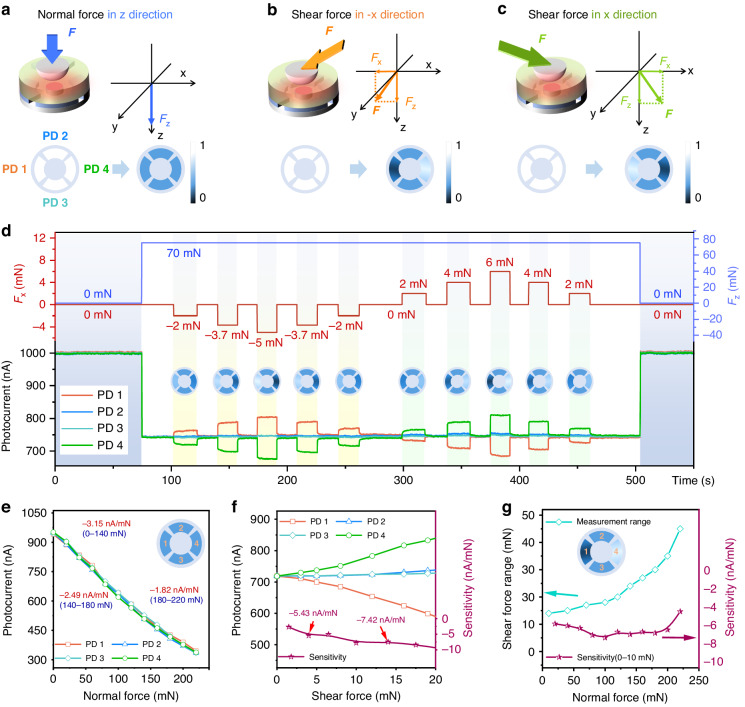
Force response characterizations of PDMS hemispherical devices. **a-c** Schematic diagrams of the applied normal force and shear force in the device, along with the normalized topographic evolution of the photocurrents received by the four PDs. **d** Response characteristic curves of hemispherical structural devices under various normal and shear forces, accompanied by the normalized topographic evolution of the photocurrents received by the four PDs. **e** Relationship curve between normal force and Photocurrent as a function of normal force variation. **f** Photocurrent as a function of Relationship curve between shear force and photocurrent variation. **g** Under different normal pressures, the response sensitivity and range of shear force

Figure 4a-c plot schematic diagrams of the applied normal force (in *z*-direction) and shear force (in *x*-direction), as well as the normalized topographic evolution of the photocurrents received by the four PDs. The values of the normal and shear forces measured by two force gauges, along with the photocurrents received by four PDs, are shown in Fig. 4d. At zero pressure, the normalized photocurrents received by the four PDs were all ~1.1 µA. During the test process, the normal pressure was applied by changing the displacement in the *z*-direction. Then, the displacement was changed in the +*x* direction (+normal, + shear) to apply shear force. Subsequently, displacement was applied in the - *x* direction while maintaining a constant normal force (+normal, - shear). After each application of shear force, it was kept constant for 20 s, then the shear force was removed, and another shear force test was conducted after a 20 s waiting period. When a normal pressure F_z_ of 70 mN along the vertical direction (*z*-axis) was applied, the photocurrents of the four PDs decreased to 0.73 μA. When a shear force was applied in the + *x*-axis direction while maintaining a constant normal pressure, the photocurrents of PD 1 and PD 4, which were respectively in the positive and negative directions (±*x*) changed in opposite ways: the photocurrent of PD 4 decreased, while that of PD 1 increased. However, PD 2 and PD 3 in the *y*-axis direction perpendicular to the *x*-axis showed minor changes. The photocurrent response curves in Fig. 4d indicated that the device exhibited a significant response to a small shear force of 2 mN and immediately recovered each time the shear force and normal force were removed, demonstrating excellent repeatability. To study the response performance of the device to vector force in different directions, a shear force was applied in each direction for testing. The device exhibited real-time and accurate responses to shear forces in eight directions within the *x*-*y* plane, spaced at 45° intervals, which was provided in Supplement 1, Fig. S3. It could be concluded that this sensor based on the PDMS hemispherical structure exhibited high sensitivity, fast response, and high repeatability.

Quantitative Characterizations of the normal force response characteristics of the device were conducted. The response characteristics were tested by applying normal forces in the *z*-axis direction using the three-dimensional displacement stage. The photocurrent responses of the four PDs of the sensor were measured and depicted in Fig. 4e. The variation trends of the four PDs were generally consistent due to the similar deformation of the PDMS hemispherical structure in all directions of the *x*-*y* plane. However, the response range of the sensor depended on the contact area between the PDMS and sapphire which altered the light intensity received by PDs. The relationship between the change in contact area and applied force was nonlinear, resulting in nonlinear response characteristics of the sensor. This nonlinearity could be alleviated in the future by improving the shape of the PDMS microstructure. Figure 4e displays the photocurrents of the four PDs as a function of the normal force. The sensitivity of the sensor in the range of 0–140 mN is −3.15 nA/mN, in the range of 140–180 mN is −2.49 nA/mN, and in the range of 180–220 mN is −1.82 nA/mN.

To measure shear force, it was necessary to ensure that PDMS hemispherical structure undergoes shear deformation under the applied force. Therefore, a certain normal pressure was applied to create friction between the PDMS and the sapphire interface, preventing sliding. By maintaining a constant normal pressure and changing the shear force, the response characteristics of the shear force were measured. The photocurrents of the four PDs as a function of the shear force are depicted in Fig. 4f, where the normal force is 70 mN. When shear force was applied in the direction towards PD 1, the reflected light intensity received by PD 1 decreased, resulting in a decrease in photocurrent. Conversely, PD 4, located in the opposite direction, received an increase in reflected light intensity, leading to an increase in photocurrent. PD 2 and PD 3 showed minimal changes in photocurrent. The device exhibits a sensitivity of −5-8 nA/mN.

Subsequently, we tested the response of the shear force under different normal pressures. More test results can be found in Supplement 1, Fig. S4. The sensor still responds under larger forces, but it loses practical value due to the sharp increase in drift and the decrease in sensitivity. Taking the shear force response under a normal force of 70 mN as an example (Supplement 1, Fig. S5), when the shear force exceeds 15 mN, the non-linear effects increase sharply, and the output photocurrents of PD 2 and PD 3 in the direction perpendicular to the shear force experience severe drift, causing the sensor to fail to correctly decouple the normal force and shear force. We tested the effective range of the sensor under different normal forces and plotted the sensitivity of shear force within the response range (0–10 mN) in Fig. 4g. The range of the shear force gradually increases with the increase in normal force. Although its sensitivity shows significant non-linearity, it maintains a high sensitivity of 5–8 nA/mN. This demonstrates that the use of this hemispherical structure for the sensor is a feasible solution.

The minimum resolution of the SMU for current is 50 pA. Under our measurement conditions, the noise fluctuation range of the sensor is ±0.5 nA. Therefore, for the hemispherical structure, even within the measurement range of the sensor’s lowest normal force sensitivity (−1.82 nA/mN) and lowest shear force sensitivity (−5 nA/mN), The sensor can distinguish normal force and shear force as low as 1 mN.

### The vector force sensing characteristics of packaged devices

**Fig. 5:**
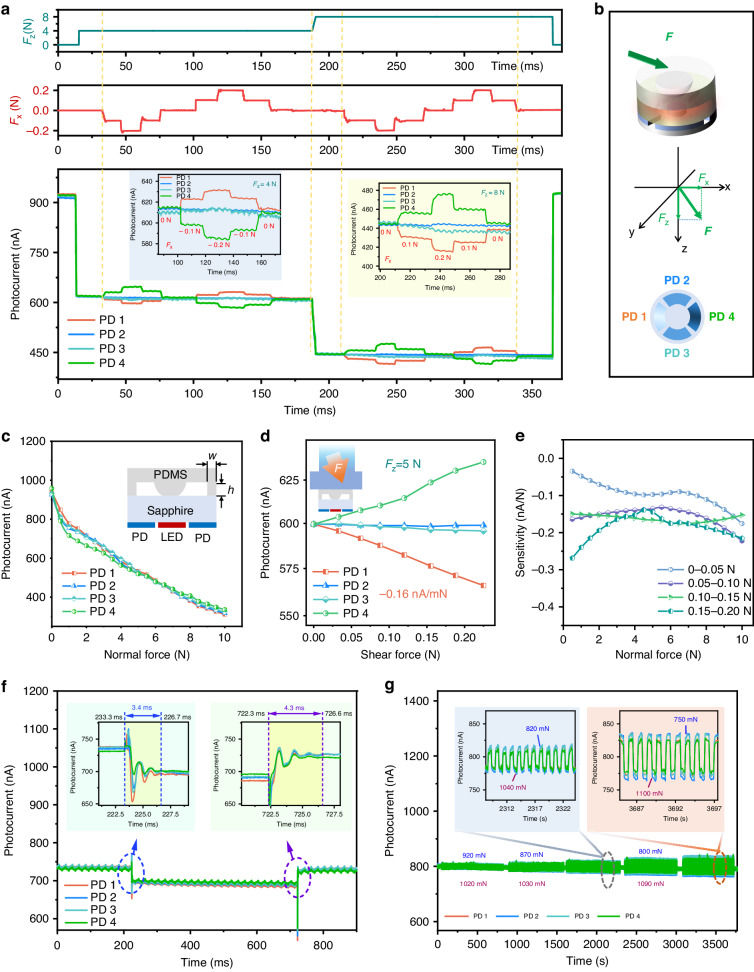
Force response characterizations of packaged devices. **a** Response of the sensor to different normal and shear forces. **b** Schematic diagrams of the applied normal force and shear forces in *x-z* direction. **c** Photocurrent as a function of normal force. **d** Photocurrent as a function of shear force, under a normal force of 5 N. **e** Sensitivity of shear force response under various normal forces. **f** Response time test of the sensor. **g** Repeatability test of the sensor

The vector force sensor was finally packaged on a cover-like PDMS structure that included the hemispherical structure. The dimensions of the PDMS part of the packaged device we fabricated were illustrated in the inset of Fig. 5c. The circular ring structure supporting the PDMS film had a width (w) of 1 mm and a thickness (h) of 0.5 mm.

The response characteristics to vector forces were measured. Figure 5a illustrates an example of the response characteristics of the vector sensor to various normal force and shear forces. As shown in Fig. 5a, when a normal pressure of 4 N in the *F*_z_ direction was applied to the sensor, the photocurrents of all four PDs changed equally to 600 nA. On the other hand, when shear forces of 0.1 N were applied in horizontal directions ( ± x), the sensor exhibited good shear force recognition capability. Upon increasing the normal pressure to 8 N, the photocurrent changes of all four PDs simultaneously decreased to 400 nA. Under a constant normal force of 8 N, applying shear forces of different directions and magnitudes, the sensor still showed a good response, but PD 2 and PD 3 began to exhibit some drift at a shear force of 0.2 N. Removing the shear force and normal pressure, the sensor quickly recovered.

In our sensor, the response to forces in the horizontal and vertical directions can be conveniently decoupled. This is because horizontal forces cause isotropic deformation of the structure. The deformation in the horizontal direction will invariably increase the contact area in the positive direction and decrease it in the negative direction, resulting in one corresponding PD photocurrent decreasing and the other increasing. Therefore, when analyzing the four photocurrent signals output by four PDs, the parts with the same sign (positive or negative) of the change (∆*I*_*i*_) correspond to the normal force information, while the parts with opposite signs of ∆*I*_*i*_ correspond to the shear force information. In Fig. 5a, when applying a normal pressure of 4 N, the photocurrents of all four PDs simultaneously decreased. With a ∆*I*_*i*_ value of −300 nA. While maintaining the normal pressure, when applying a 0.1 N shear forces along the -*x* direction, it could be observed that the photocurrent of PD 1 decreased (∆*I*_1_ = −11.3 nA), while the photocurrent of PD 4 increased (∆*I*_4_ = +10.4 nA). At the same time, the photocurrents of PD 2 and PD 3 remained almost unchanged (∆*I*_2_, ∆*I*_3_≈0). Therefore, based on the relative changes in photocurrents of the four PDs, detailed information about the normal and shear forces could be easily recognized.

The characterization of the sensor’s vector force response characteristics was conducted using the same method as in the previous section. Figure 5c presents the relationship between the sensor’s output photocurrent and the applied normal force. There is a significant error in the 0–2 N range, mainly due to fabrication errors. Within the 0–1 N range, the sensor exhibits a sensitivity of −0.19 nA/mN. In the 1–5 N range, the sensitivity is −0.06 nA/mN. In the 5–10 N range exhibits a sensitivity of −0.04 nA/mN. Subsequently, we tested the sensor’s response characteristics to shear force. Figure 5d shows the sensor’s shear force response characteristics under a normal force of 5 N. In the 0–0.2 N range, the sensor has a good linear response with a sensitivity of −0.16 nA/mN. We conducted separate tests for different normal forces and shear forces, and more test results can be found in Supplement 1, Fig. S6. The relationship between the normal force and sensitivity of the sensor is depicted in Fig. 5e. Under smaller normal forces, the response sensitivity to shear forces in the 0–0.2 N range exhibits strong nonlinearity. As the normal force increases, the linearity of sensitivity improves.

We tested the response time of the sensor using a piezoelectric element to rapidly apply and remove force by displacing a force gauge probe. As shown in Fig. 5f, the response time of the sensor to pressure is 3.4 ms, and the recovery time is 4.3 ms. It is noteworthy that the signal exhibits significant fluctuations after the response, primarily due to the inertia of the force gauge probe. Finally, we tested the repeatability of the sensor by conducting 500 cycles of tests with five sets of varying normal forces, as shown in Fig. 5g. The sensor’s response remained stable in each cycle, demonstrating the good repeatability of our sensor. Considering the measurement accuracy of the SMU and environmental noise as well, the sensor can detect a minimum normal force of 25 mN and a minimum shear force of 20 mN, with a measurement range of 10 N for normal force and 0.2 N for shear force.

### Surface texture recognition validation

The sensors were further encapsulated with copper conical probes, and the functionality of the sensor in texture recognition was verified. Subsequently, texture recognition tests were conducted on samples featuring various patterns of embossed textures.

**Fig. 6:**
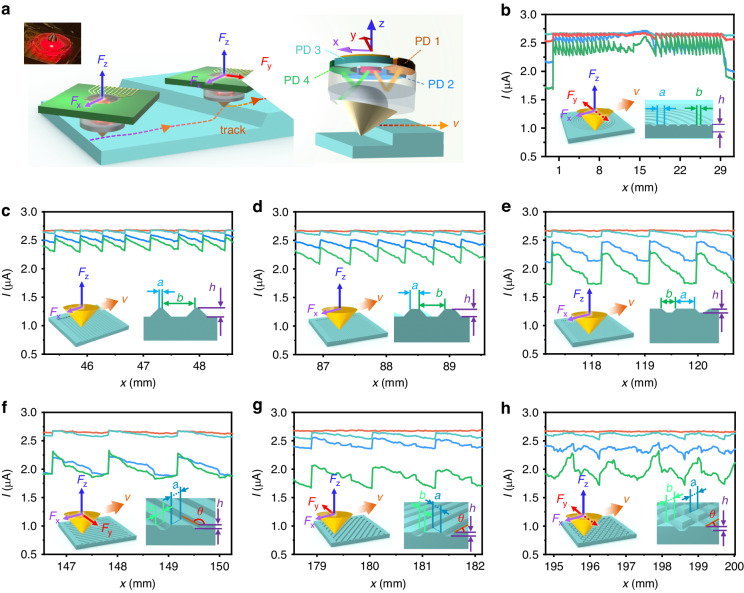
Demonstrative experiments of surface texture recognition. **a** Demonstrative experiments of surface texture recognition. inset is the camera image of the sensor. The magnified image indicates the orientation of the internal PDs of the sensor with respect to the coordinate axes. Characteristic of signals measured for several texture patterns, **b** circular, **c**–**e** straight stripe, **f**, **g** diagonal, **h** rhombic, inset shows the force schematic of the sensor with a schematic of the texture cross-section

Following the surface texture sensing strategy of human fingers, we conduct the texture recognition test by sliding our sensor on the surface of an object. As shown in Fig. 6a, the magnitude and direction of the force on the probe will be changed with the topography of the surface, as the sensor probe slides on the sample surface in PD 1 direction (-x direction). Therefore, by analyzing the photocurrents of the four PDs on the sensor, the force applied to the probe could be obtained, which in turn could distinguish the texture pattern of the sample surface. Seven textures, including concentric rings, fringes with different spacing, twill lines with different orientations, and rhombus shapes, were tested for recognition. Figure 6b–h depicts the corresponding photocurrent signals of these samples, and the insets show a corresponding schematic diagram of the force applied to the probe during the testing process and a schematic diagram of the cross-section of the textured samples. It can be seen that the sensor signals are significantly different for various texture patterns, indicating substantial potential for the fast recognition of texture patterns through the proposed monolithic integrated micro-scale vector force sensor.

## Discussion

In this study, we achieved a real-time measurement vector force sensor with highly sensitive and rapid responsiveness by employing a monolithic integrated optoelectronic chip. The design incorporates a monolithic sapphire-based AlGaInP optoelectronic chip integrated with a micro-hemispherical PDMS structure. This sensor can real-time detect the magnitude and direction information of force including normal forces and shear forces.

The success of realizing a compact sensor depends on using the chip substrate directly as an optical coupling component in our device, which involves a lift-off and bonding process, as well as replacing the chip substrate with sapphire. The optical coupling efficiency of the device can be enhanced by finely adjusting the thickness of the sapphire substrate through precise grinding and polishing techniques. Moreover, the AlGaInP chip fabrication is a mature and low-cost technology. The sensor can operate even at the injection current of 0.1 mA, and the stable emission properties of such micro-LED, ensure consistent and reliable performance over an extended duration, enabling the sensor to maintain stable operation while consuming minimal power. The packaged sensor, with only PDMS and sapphire surfaces in contact with the external environment, ensures long-term stable operation of the device even in harsh environments, due to the excellent stability exhibited by both materials. Additionally, our micro-LED is AlGaInP red micro-LED, operating in the biologically friendly red-light range (600–670 nm), ensuring the sensor’s applicability to biocompatible fields.

In addition to these forward-looking research directions, technical improvements and extensions should also be considered. With advancements in micro- and nanofabrication technologies, it is hopeful that high-performance sensors with smaller units can be produced, further improving sensor integration and reducing manufacturing costs. The sensitivity and range of the sensor largely depend on the mechanical properties of the central hemisphere and the packaging structure. This can be adjusted to suit different application scenarios by selecting appropriate materials and optimizing the structure. For example, optimizing the structure of the central hemisphere through mechanical simulation, adjusting the shape of the hemisphere to an elliptical or conical shape, and replacing the support part with a rigid material with much less creep than PDMS as a spring structure, are expected to provide better linear responsiveness over a wider range. On the other hand, adopting different packaging structures can adjust the range and sensitivity of the sensor, but increasing the range and sensitivity are contradictory. In actual applications, it is necessary to select the appropriate structure based on the requirements. For applications requiring large-range measurements, hard materials can be used as the support structure to share more force. For applications requiring high sensitivity in a small range, a protruding center packaging structure can be used to transfer more pressure to the hemisphere structure, achieving high-sensitivity sensing. By changing the PDMS hemisphere support film to a Permalloy alloy film or a metal mesh structure, external electromagnetic interference can be shielded to improve the signal-to-noise ratio for use in scenarios with severe electromagnetic interference.

In summary, we have demonstrated a micro vector force detection sensor with high sensitivity to vector force and rapid millisecond-level responsiveness, achieved through a monolithic integrated approach. The packaged sensor can detect a minimum normal force of 25 mN and a minimum shear force of 20 mN, with a measurement range of 10 N for normal force and 0.2 N for shear force. By optimizing the sensor’s structure, it is expected that the detection limit of this hemispherical structure device can approach 1 mN. This device is capable of detecting normal and shear forces as low as 1 mN within a measurement range of 0–220 mN for normal force and 0–15 mN for shear force, with a millisecond-scale response time. This high-performance sensor exhibits several advantageous features, including a small footprint of approximately 1.5 mm², cost-effectiveness, and high stability. These attributes underscore its significant potential in various applications in pressure sensing-related domains, such as surface texture tactile recognition. Furthermore, such a sensor can be effectively integrated with other functional devices, enabling real-time simultaneous detection of more comprehensive sensing information, thereby enhancing its capabilities and expanding its potential applications in robotic surgery and smart wearable equipment.

## Methods

### Simulation of the FEA

Finite Element Analysis (FEA) was conducted utilizing the commercial software Abaqus, with a simplified two-dimensional model. The PDMS was represented in the model as an incompressible neo-Hookean material, with a Young’s modulus (*E* = 2.4 MPa) determined through experimental measurements. The evolution of the contact area was tracked as the normal force gradually increased to 70 mN, followed by an increase in shear force up to 20 mN.

### Simulation of Ray-tracing

The metal electrode features were omitted to reduce the computation time. The top and bottom surfaces of the MQW layer in the LED were selected as light sources, each with a light power of 1 W and 50,000 rays. The distance between the PD and the LED was set at 200 μm, with a sapphire layer thickness of 340 μm.

### The fabrication process of AlGaInP optical chip

The AlGaInP/sapphire device, which including a central micro-LED and four PDs, was fabricated on an AlGaInP-on-sapphire wafer containing AlGaInP MQW utilizing wafer-level microfabrication techniques. The fabrication process was described in Supplement 1, Section 2.

### Fabrication process of the hemispherical PDMS elastomer

The hemispherical PDMS elastomer is manufactured by mixing pre-polymer and curing agent (Sylgard 184, Dow Corning) in a 10:1 ratio, pouring it into a patterned PTFE mold, cured at 80 °C for 2 h, and placed on the sapphire surface of the AlGaInP/sapphire chip. A thin layer of PDMS liquid is applied and cured in situ to bond with the sapphire surface.

### Methods for surface texture recognition validation

The sensors were bonded together with copper conical probes using PDMS in-situ curing. The texture pattern samples were fabricated by computer numerical control (CNC) milling an acrylic sheet and the dimensional parameters of the textured samples are shown in Supplement 1, Table. S1. For testing, the probe and the sample were controlled by stepper motors to slide along a straight-line trajectory at a relative speed of 350 μm/s. The signals from the photocurrents on the four PDs of the sensor were acquired by a four-channel SMU at a frequency of 10 Hz. Physical drawings of the textured samples, as well as the test schematic are shown in Supplement 1, Fig. S7.

### Supplementary information

Supporting file
