# Supplementary material for: Monolithic integrated optoelectronic chip for vector force detection
Source: Microsyst Nanoeng. 2024 Jun 24;10:85. doi: 10.1038/s41378-024-00712-6 (PMC11194277; doi:10.1038/s41378-024-00712-6)
Supplement: Supplementary file 1 — Supporting file [file 41378_2024_712_MOESM1_ESM.docx]

Monolithic Integrated Optoelectronic Chip for Vector Force Detection

Jiansong Feng,^1†^ Zhongqi Wang,^2†^ Mengyuan Zhanghu,^1†^ Xu Zhang,^1^ Yong Shen,^1^ Jing Yang,^1^ Zhibin Li,^1^ Bin Chen,^1^ Taihong Wang,^1*^ Xiaolong Chen,^1*^and Zhaojun Liu^1*^

1 Department of Electrical and Electronic Engineering, Southern University of Science and Technology, Shenzhen 518055, China

2 Department of Electrical and Electronic Engineering, The University of Hong Kong, Hong Kong, China

These authors contributed equally: Jiansong Feng, Zhongqi Wang and Mengyuan Zhanghu

Corresponding author. E-mail: [wangth@sustech.edu.cn](mailto:wangth@sustech.edu.cn) (Taihong Wang), [chenxl@sustech.edu.cn](mailto:chenxl@sustech.edu.cn) (Xiaolong Chen), [liuzj@sustech.edu.cn](mailto:liuzj@sustech.edu.cn) (Zhaojun Liu).

1. **Experimental setup for vector force test**

The test setup comprises force gauges and displacement tables in both horizontal and vertical directions. The device is fixed on the stage. The stage is fixed to the horizontal force gauge probe, and rigid rollers support the base, allowing free rolling between the rollers and the stage. Adjust the displacement table knob to bring the force gauge probe into contact with the device. Either normal or shear force is applied to the device by adjusting the three-dimensional stage.

**Fig. S1** Schematic diagram of the vector force sensor characteristics testing device.

1. **Fabrication process of AlGaInP optoelectronic chip**

The AlGaInP/sapphire device was fabricated on a sapphire-based AlGaInP wafer containing AlGaInP MQW utilizing wafer-level microfabrication techniques. The fabrication process was briefly described as follows [1].

After removing the GaAs substrate and bonding to sapphire substrate, red micro-LEDs and PD devices were fabricated using sapphire based AlGaInP epitaxial wafers. The AlGaInP surface was cleaned with a solution of HCl:H_3_PO_4_ = 2:1 for 2 minutes, rinsed with deionized water, and then sequentially soaked in acetone, isopropanol, and deionized water. Silicon dioxide (2 μm) was deposited using plasma-enhanced chemical vapor deposition (PECVD) as a mask and patterned by photolithography. The mesa was formed by inductively coupled plasma (ICP) etching. A 200 nm Au/Zn alloy is deposited on the p-type GaP electrode by Electron-beam (E-beam) evaporation. Followed by annealing at 400 °C in a nitrogen atmosphere for 5 minutes to achieve ohmic contact. For the n-type GaP layer, a 200 nm Ni/Ge/Au alloy was used as the electrode, and annealing was carried out at 380 °C in a nitrogen atmosphere for 5 minutes to form ohmic contact. The radius of the central micro-LED was 238 μm. The PD had a fan-shaped dimension with an inner circle diameter of 450μm and an outer circle radius of 750 μm. The distance between the PD and micro-LED edges was 200 μm. Finally, thinning and polishing of the sapphire surface were performed, resulting in a thickness of 340 μm for the sapphire layer. The bonding wires were connected from the electrode leads to an external power supply and detection instruments using a wire bonding machine.

**Fig. S2** AlGaInP optoelectronic chip manufacturing process

1. **The response characteristics of packaged devices to vector forces in each direction.**

The packaged devices were rotated on the test stage at a 45º interval to measure the vector force response characteristics in each direction. During the measurement of the photocurrent response of the sensor under various pressure loads, the micro-LED driving current was kept constant at 400 μA, and the PDs were maintained at a bias voltage of 0 V.

**Fig. S3** Response test of the sensor to vector forces in each direction. The normal force is kept constant throughout the test, and only the magnitude and direction of the shear force is changed.

1. **The response of shear force for the sensor with hemispherical structure under various normal pressures.**


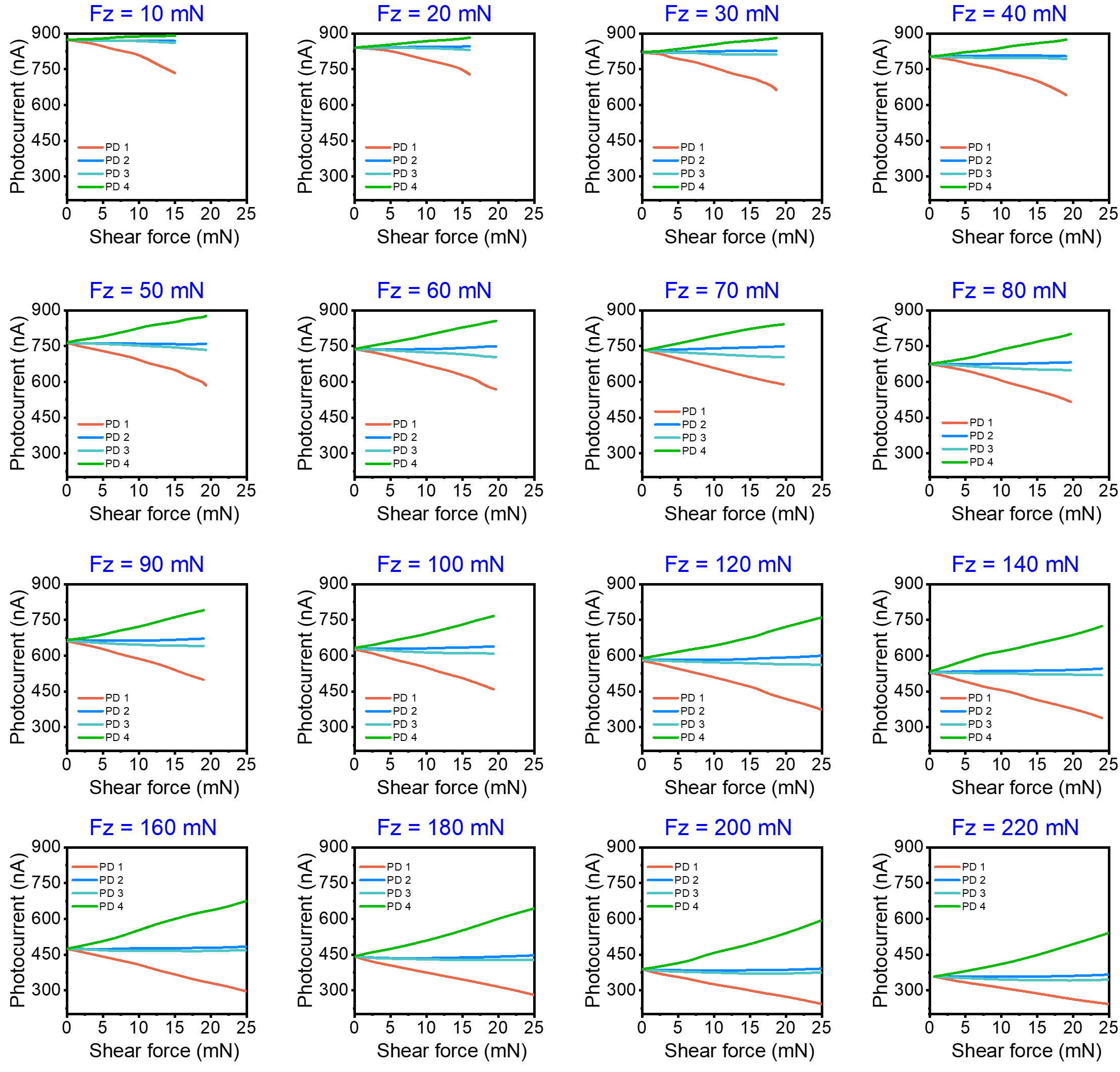


**Fig. S4** The response of shear force for the sensor under various normal pressures ranging from 10 to 220 mN, using the hemispherical structure.

**5. The method for determining the shear force range of the sensor.**

Taking the response under a normal pressure of 100 mN as an example, as shown in the figure below, the sensor has a good response within the 0-15 mN shear force measurement range. However, beyond 15 mN, PD 2 and PD 3 begin to drift. Although the sensor still has a good linear response to shear force in the range of 15 to 50 mN (as shown by PD 1 in the figure), due to the drift error of PD 2 and PD 3, it is not possible to accurately decouple the shear force and normal force. Therefore, the range of the device under a normal pressure of 100 mN is determined to be 0-15 mN.

**Fig. S5** Experimental test of the shear force range.

**6. The response of shear force under various normal pressures for the encapsulated device.
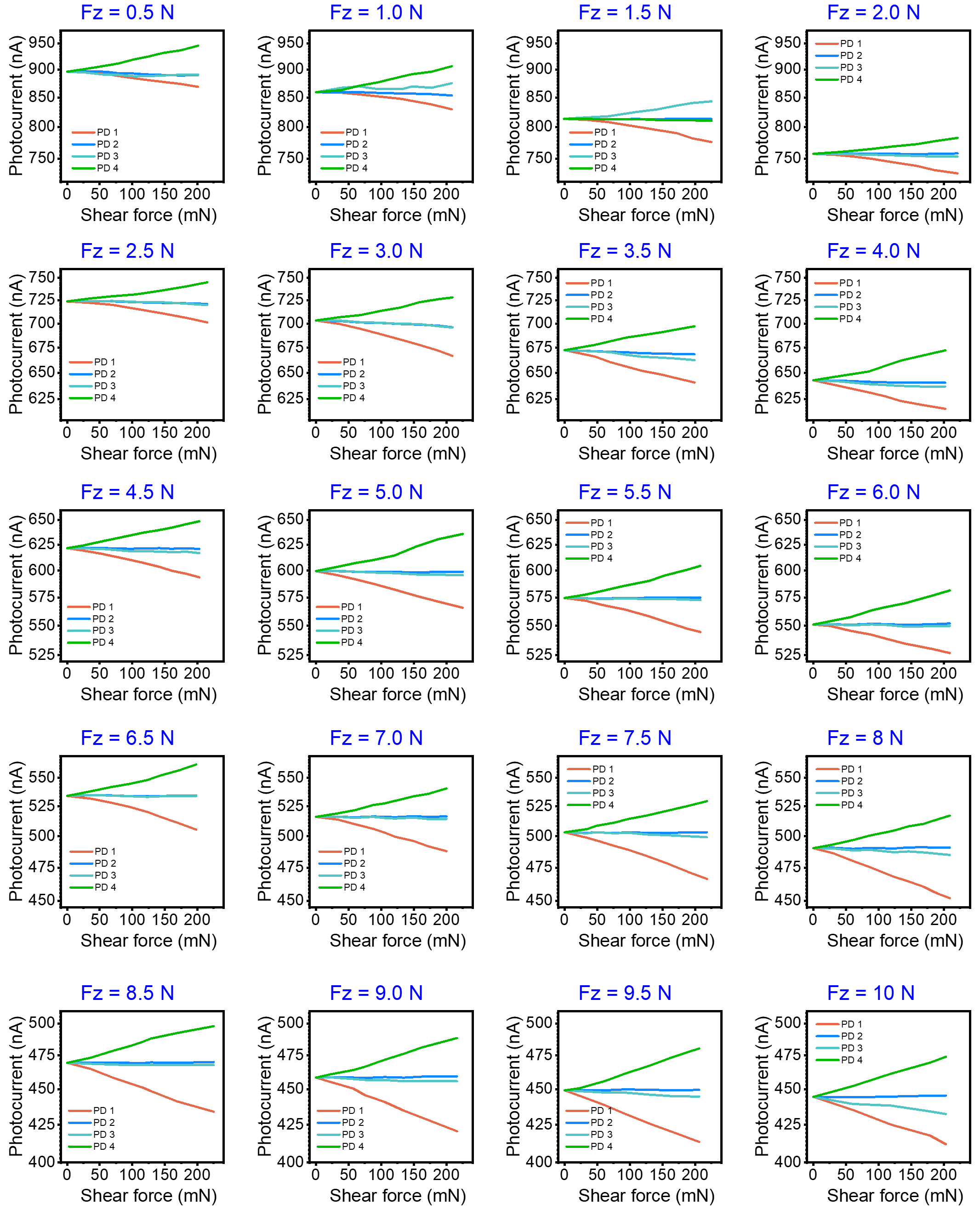
**

**Fig. S6** Results of the packaged sensor's response to shear forces ranging from 0 to 0.2 N under normal forces of 0.5 to 10 N.

1. **Surface texture recognition validation**

**Fig. S7** Global signals for texture recognition tests. The inset shows optical microscope images of seven texture sample cross-sections.

Table. S1 Measured dimensional parameters of various textured samples

| Sample | Texture | a (μm) | b (μm) | h (μm) | θ (°) |
| --- | --- | --- | --- | --- | --- |
| 1 | Circle | - | - | 153 | 0~180 |
| 2 | Fringes | 22 | 481 | 122 | 0 |
| 3 | Fringes | 32 | 475 | 181 | 0 |
| 4 | Fringes | 386 | 614 | 238 | 0 |
| 5 | Twill line | 451 | 542 | 183 | 145 |
| 6 | Twill line | 452 | 522 | 151 | 45 |
| 7 | Rhombus | 557 | 441 | 149 | 45, 145 |

**Reference**

1. M. Zhanghu, W. Huang, H. Yang, *et al*., “P-8.1: High-performance AlGaInP Based Red Micro-LED,” SID Symposium Digest of Technical Papers **53**, 874-876, (2022).
